# Metastatic Bladder Cancer Presenting with Persistent Hematuria in Young Man with Cystic Fibrosis

**DOI:** 10.1155/2013/831871

**Published:** 2013-05-14

**Authors:** Premal Patel, Harvey R. Rabin, Michael M. Vickers, Michael D. Parkins

**Affiliations:** ^1^Department of Medicine, The University of Calgary, Calgary, AB, Canada T2N 4N1; ^2^Department of Microbiology and Infectious Diseases, The University of Calgary, Calgary, AB, Canada T2N 4N1; ^3^Department of Oncology, The University of Calgary, Calgary, AB, Canada T2N 4N1; ^4^3330 Hospital Drive NW, Health Sciences Center, The University of Calgary, Calgary, AB, Canada T2N 4N1

## Abstract

We report a case of metastatic bladder cancer developing in a young man with cystic fibrosis (CF) that was initially diagnosed as ureterolithiasis and managed as renal colic. With the improved survival of patients with CF, an increasing burden of extrapulmonary disease manifestations is apparent. Renal colic is observed at an increased frequency in patients with CF relative to the general population and is a commonly recognized cause of hematuria. However, CF patients harboring a malignancy are recognized to be at increased risk of delayed identification owing to atypical symptoms and lack of demographic risk factors. This case illustrates how investigations to rule out malignancy are warranted in those CF patients not responding to therapies directed towards presumptive diagnoses.

## 1. Introduction 

Innovations in screening, pharmacotherapy, and management have reduced the morbidity and mortality of patients with cystic fibrosis (CF). From 1985 to 2010, the proportion of individuals with CF achieving adulthood (>18 years of age) increased from 29.9% to 57.2%, and median survival age increased from 36 years in 2000 to 48.1 years in 2010 [1]. With improvements in survival, increasing age-related CF complications such as malignancy must be followed [2].

## 2. Case Presentation

This 29-year-old male was diagnosed with CF (F508del/F508del) at two months of age. His CF is notable for pancreatic insufficiency, moderate airways disease with chronic *Pseudomonas aeruginosa* infection, severe CF related liver disease, and diabetes. His medical regimen included twice daily physiotherapy with a flutter, and he was maintained on Creon, ADEKs, vitamin D, Ventolin, Flovent Diskus, Pulmozyme, tobramycin inhalation solution (TOBI), Urso falk, Colace, and Nasacort.

**Figure 1:**
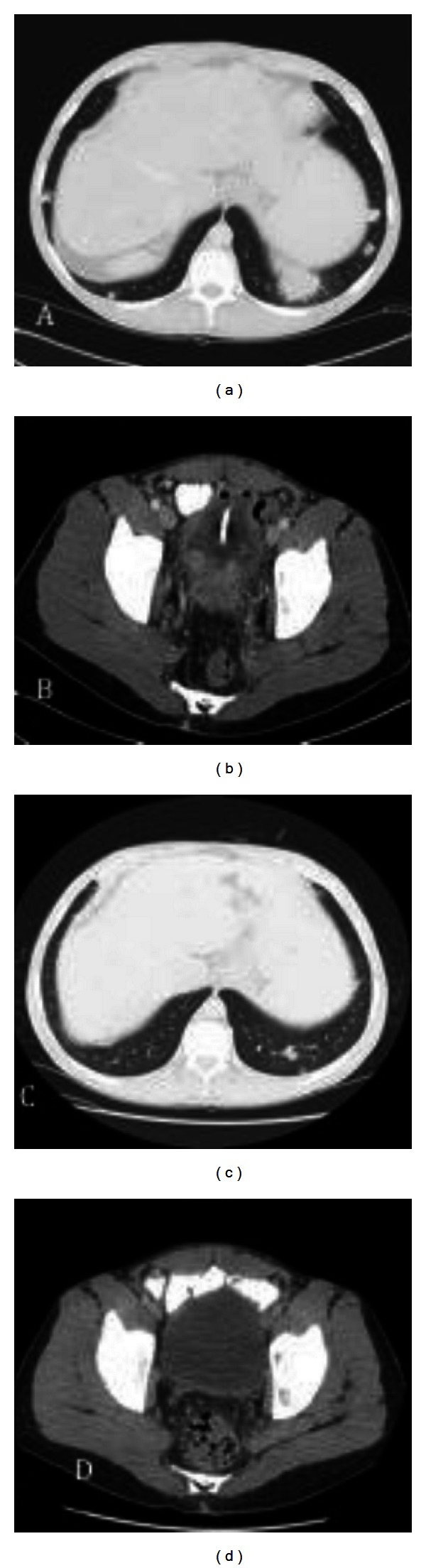
CT scans showing dominant pulmonary nodule in left lower lobe and bladder thickening, pre-chemotherapy (panels A and B), and two months after completion of chemotherapy (panels C and D). These scans demonstrate the stability of the pulmonary nodules, and resolution of the pelvic mass and lymphadenopathy and his subcutaneous nodules.

He presented with asymptomatic hematuria, at which time a CT scan demonstrated a calculus that was treated with a ureteric stent. Diagnostic cystoscopy was unremarkable. Hematuria persisted, requiring an admission two months later with a presumptive diagnosis of prostatitis. Repeated cystoscopies failed to identify new pathology. After an initial improvement in symptoms, five months after initial presentation he developed new scrotal/anal pain. Finally, a fifth cystoscopy was undertaken and revealed a mass overlying the right ureteric orifice and prostate. A transurethral resection was performed and pathologic assessment revealed an invasive high-grade urothelial carcinoma. Immunohistochemical staining was positive for vimentin and cytokeratin 5/6 and negative for PSA and neuroendocrine markers. A staging CT scan revealed evidence of both local invasion into adjacent structures in the pelvis regional, skin nodules and metastatic spread (Figure 1). A lifelong non-smoker, he did not consume alcohol and lacked occupational risk factors for bladder cancer. Prior radiation exposure was also minimal. 

Initial management included cisplatin and gemcitabine, at a 25% dose reduction due to baseline thrombocytopenia. After completing six cycles of chemotherapy, a CT scan showed partial response (Figure 1). The patient tolerated chemotherapy well and required only one red blood cell and platelet transfusion. However, at 11 months from initial presentation he experienced new-onset left groin pain. A repeat CT scan showed bilateral iliac lymphadenopathy and new boney metastases. After six additional cycles of therapy, he demonstrated a decrease in the size of the pulmonary nodules and pelvic lymph nodes and resolution of local pain. Sixteen months after initial presentation, he developed severe lower back pain. A CT scan at that time demonstrated further progression of his disease now involving his lung parenchyma, mediastinal, hilar, and periaortic lymph nodes and multiple bony lesions.

The patient succumbed to his metastatic bladder cancer almost thirty months after his initial presentation at the age of 31. 

## 3. Discussion

While studies have conclusively reported that gastrointestinal (GI) cancers in CF occur at a rate of 5.1–6.5 [3–6] times that of the general population, there are conflicting reports as to whether there is an overall increased risk of other cancers [3, 5, 6]. Other notable malignancies that may be observed at an increased incidence in CF include kidney, thyroid, endocrine, lymphoma, and nonmelanoma skin cancers [6]. Bladder cancer has been reported to affect another patient with CF [5]; however, details are lacking. 

Our patient lacked all recognized risk factors for bladder cancer [7]; his age was less than half the median age of patients with bladder cancer (69 years for males) [8], and he experienced a more aggressive disease than expected for his age [9]. This disproportionally early onset of cancer is common among CF-associated malignancies. Neglia et al. conducted a prospective cohort study which assessed malignancy risk among over 38, 000 CF individuals [3]. This study found that the average age of onset of GI malignancies among CF patients was 32.2 ± 12.6 years, compared to 58.2 ± 14.3 years of age in control populations. 

CF patients suffer a variety of extrapulmonary disease-related clinical manifestations that are increasingly apparent with advancing age [2, 10], including renal colic. A recent study found the prevalence of nephrolithiasis to be 21% among 29 CF patients aged 28.4 ± 7.1 years as opposed to 0 for the control group (30 patients aged 39.1 ± 11.5 years) [11]. As nephrolithiasis and malignancy in CF patients typically occur at a younger age, it is prudent to assess for both. There is limited data to define the influence of CFTR expression and the development of bladder cancer; however, a recent report by Yu et al. suggests that identification of hypermethylated CFTR in the urine may aid in the detection of bladder cancer [12]. There is no evidence to suggest that his CF made the detection of the bladder cancer more difficult, requiring five cystoscopies before a lesion could be positively identified. 

In the management of CF patients with malignancy, concerns may arise regarding the benefit and toxicity of systemic therapies. This is the first case to specifically report a response to chemotherapy without significant tolerability issues in this patient population. CF patients are recognized as having an altered drug disposition relative to non-CF patient populations, typically related to a larger volume of distribution, and a greater total body clearance [13, 14]. While an abundance of pharmacokinetic data exists to guide altered drug dosing for CF patients receiving antimicrobial agents, there is no data to suggest that chemotherapeutics may need similar alterations. Von der Maase et al. established cisplatin and gemcitabine as a standard treatment for advanced bladder cancer and reported a median time to progression of 7.4 months, similar to that observed herein [15]. Acceleration of pulmonary function decline and precipitation of pulmonary exacerbation are also a concern for CF patients chronically colonized with respiratory pathogens. Like other case reports of CF patients requiring novel cytotoxic and immunosuppressive therapies, our patient did not experience a significantly higher requirement for antibiotics during chemotherapy [16, 17]. 

## 4. Conclusion

This case underscores the importance of considering malignancy risk in CF patients, especially those with abnormal presentations that do not respond to treatment. To our knowledge, this is the first detailed case report of bladder cancer in a CF patient and the first to report on response to therapy. Our experience suggests that cytotoxic chemotherapy may be well tolerated in CF patients; this patient population can potentially benefit from this therapy. As medical interventions continue to improve, so will the median survival of this population. Health care providers need to meet the current and future needs of this population.

## References

[1] Foundation CCF (2007). *Canadian Cystic Fibrosis Foundation Patient Data Registry Report*.

[2] Parkins MD, Parkins VM, Rendall JC, Elborn S (2011). Changing epidemiology and clinical issues arising in an ageing cystic fibrosis population. *Therapeutic Advances in Respiratory Disease*.

[3] Neglia JP, FitzSimmons SC, Maisonneuve P (1995). The risk of cancer among patients with cystic fibrosis. *New England Journal of Medicine*.

[4] Schoni MH, Maisonneuve P, Schoni-Affolter F, Lowenfels AB (1996). Cancer risk in patients with cystic fibrosis: the European data. CF/CSG Group. *Journal of the Royal Society of Medicine*.

[5] Maisonneuve P, FitzSimmons SC, Neglia JP, Campbell PW, Lowenfels AB (2003). Cancer risk in nontransplanted and transplanted cystic fibrosis patients: a 10-year study. *Journal of the National Cancer Institute*.

[6] Johannesson M, Askling J, Montgomery SM, Ekbom A, Bahmanyar S (2009). Cancer risk among patients with cystic fibrosis and their first-degree relatives. *International Journal of Cancer*.

[7] Murta-Nascimento C, Schmitz-Dräger BJ, Zeegers MP (2007). Epidemiology of urinary bladder cancer: from tumor development to patient’s death. *World Journal of Urology*.

[8] Lynch CF, Cohen MB (1995). Urinary system. *Cancer*.

[9] Wen YC, Kuo JY, Chen KK (2005). Urothelial carcinoma of the urinary bladder in young adults—Clinical experience at Taipei Veterans General Hospital. *Journal of the Chinese Medical Association*.

[10] Perez-Brayfield MR, Caplan D, Gatti JM, Smith EA, Kirsch AJ (2002). Metabolic risk factors for stone formation in patients with cystic fibrosis. *Journal of Urology*.

[11] Terribile M, Capuano M, Cangiano G (2006). Factors increasing the risk for stone formation in adult patients with cystic fibrosis. *Nephrology Dialysis Transplantation*.

[12] Yu J, Zhu T, Wang Z (2007). A novel set of DNA methylation markers in urine sediments for sensitive/specific detection of bladder cancer. *Clinical Cancer Research*.

[13] Touw DJ (1998). Clinical pharmacokinetics of antimicrobial drugs in cystic fibrosis. *Pharmacy World and Science*.

[14] Rey E, Tréluyer JM, Pons G (1998). Drug disposition in cystic fibrosis. *Clinical Pharmacokinetics*.

[15] Von der Maase H, Hansen SW, Roberts JT (2000). Gemcitabine and cisplatin versus methotrexate, vinblastine, doxorubicin, and cisplatin in advanced or metastatic bladder cancer: results of a large, randomized, multinational, multicenter, phase III study. *Journal of Clinical Oncology*.

[16] Casserly B, Donat W (2009). Stabilization of lung function and clinical symptoms in a patient with Cystic Fibrosis (CF) after institution of infliximab: a monoclonal antibody that binds tumor necrosis factor alpha. *Lung*.

[17] Vincenzi F, Bizzarri B, Ghiselli A, de’ Angelis N, Fornaroli F, de’ Angelis GL (2010). Cystic fibrosis and Crohn’s disease: successful treatment and long term remission with infliximab. *World Journal of Gastroenterology*.

